# Combination of gamma irradiation and storage condition for improving mechanical and physical postharvest characteristics of fresh garlic cloves

**DOI:** 10.1002/fsn3.3186

**Published:** 2022-12-21

**Authors:** Seyedeh Hoda Yoosefian, Ebrahim Ahmadi, Ayat Mohammad‐Razdari

**Affiliations:** ^1^ Department of Biosystem Engineering, Faculty of Agriculture Bu‐Ali Sina University Hamadan Iran; ^2^ Department of Mechanical Engineering of Biosystems Shahrekord University Shahrekord Iran

**Keywords:** discrimination, gamma irradiation, garlic, multivariate computational approaches, optimization

## Abstract

The aim of this study was the discrimination and optimization of irradiation effect under physical and mechanical experiments on garlic. The samples were irradiated with 0, 75, and 150 Gy doses and stored at 4 and 18°C for 5 months. Physical, mechanical, and color properties were measured in the period of storage. Based on the results, all irradiated garlic samples had less quality variation than control samples. Response surface methodology (RSM) optimized dose, storage time, and temperature of the stored garlic which was 75 Gy, 2 months, and 17°C, respectively. In addition, after finding the optimal dose, time, and temperature, the most effective factor as weight loss was obtained and the data were classified by the principal component analysis (PCA) approach. The results showed that the PCA method had a high ability to classify and separate the data obtained from measuring the physicochemical properties of garlic and cover 99% variance of data. Moreover, partial least square (PLS) was applied for predicting weight loss data with R2 0.9999. As well, a mechanical test was investigated for finding the best situation and duration of storage condition. Finally, irradiation prevented the destruction of garlic and saved garlic in the best quality as compared with control or nonirradiated samples. After all this, it can be decided to keep garlic in warehouses and transfer this product with minimum damage.

## Graphical abstract

Irradiated garlic storage for 5 months under controlled condition. Optimization of effective parameters was prepared by RSM. Discrimination of irradiated garlic was done by chemometric approach. Mechanical test was completed for storage situation and duration.

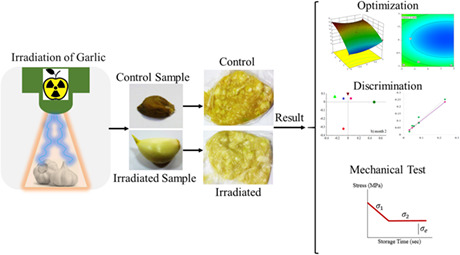

## 1 INTRODUCTION

Preservation of garlic as an export basic crop for a long time is very important. Garlic is rich in folic acid, vitamin C, calcium, iron, magnesium, potassium, zinc, and vitamins B_2_, B_1_, and B_3_ (Balmori et al., 2019; Eugster et al., 2018). It is necessary to maintain the quality of this type of food that is rich in nutrients for a long time (Noda et al., 2018). In order to preserve foods, there are different ways, such as thermal, freezing, drying, fermentation, coating, packaging film, irradiation, atmosphere control, etc. (Li et al., 2016).

Irradiation is an advanced technical approach to preserve the physical and chemical properties of the food product (Bearth & Siegrist, 2019; Güler et al., 2017). Gamma irradiation is a nonthermal method and has emerged for the preservation of fruits and vegetables to prevent the use of chemical preservatives (Olanya et al., 2015). Many studies have described the useful effect of low‐dose irradiation (50–150 Gy) for sprouting inhibition (Zhang et al., 2016). Some studies showed that storage of irradiated garlic with 100 Gy dose in a refrigerator environment reduces weight loss and spoilage (Martins et al., 2016).

Designing experiments using response surface methodology (RSM) was performed for the first time in 1950. It was initially used for chemical industries, but recently, RSM has been widely used to improve quality, design product, and analyze uncertainty. RSM is a set of statistical and applied mathematics techniques to build experimental models. The aim of this type of design is to optimize the response (output variable) which is influenced by several independent variables (input variables) (Mohammad‐Razdari et al., 2021).

To better understand the data of chemistry, the application of statistical sciences, computers, mathematics, and graphics is necessary. Chemometrics methods are used to gain chemical information obtained in the laboratory, in such a way that useful information is extracted by analyzing the obtained chemical data. Based on this information, the desired experiments can be designed with high efficiency. For data analysis, researchers use PCA (principal component analysis), LDA (linear Discriminant Analysis), ANN (Artificial Neural Network), K‐NN (Nearest Neighbor), and multivariate regression (Tazi et al., 2019).

Due to the cost of keeping garlic in warehouses, limited storage environment, high garlic production in Iran, and its high consumption, it is very important to try to improve the quality of garlic. Also, irradiation is considered as an effective way to preserve garlic and improve its safety (Hassan et al., 2019). Research on the application of irradiation treatment on the garlic product has been carried out to control pests and stop microbial activity, but the effect of treatment on the quality properties, especially the mechanical properties of this product had not been done. Herein, based on our knowledge, for the first time was investigated discrimination of the results based on the chemometric approach and optimization by response surface method in gamma‐irradiated garlic.

## 2 MATERIALS AND METHODS

### 2.1 Samples preparation

Fresh garlic bulbs were harvested (30 kg) from a local place in Hamedan, Iran in 2015, and transported (15 kg of all garlic bulbs) to the Atomic Energy Organization of Iran. Fresh samples were stored to uniform size (mean 45 mm), color free from visual defects, or damages and then packed into paper bags. Samples were irradiated by γ‐ray with Gamma Cell 220 (^60^Co) with a dosage rate of 3.05 Gy/s and the irradiation dose was 0 Gy (control), 75, and 150 Gy (Fernandes et al., 2016). The nonirradiated and irradiated garlic samples were transferred to the laboratory and further divided into paper bags (three packages for each treatment as replication and 10 garlic bulbs were placed inside each paper package) and stored under two different storage conditions: (a) 18°C temperature and 65%–70% relative humidity and (b) refrigerator (second place) was with 4°C temperature and 70%–80% relative humidity.

### 2.2 Physical and chemical parameters

The garlic clove color was measured with the portable colorimeter (HP‐200, China). In addition, the humidity and weight loss of garlic cloves were investigated through storage (Borchert et al., 2014). In order to estimate the allicin, the amount of total pyruvate and nonenzymatic pyruvate were calculated. After removing the contaminants, 50 g of the sample was crushed well with 100 ml of distilled water in an electric mixer (German Yuro‐Sonic). After 10 min, the resulting solution was filtered through Erlenmeyer filter paper. From the filtered extract mixture, 50 μl was taken and poured into the tube with 2 ml of distilled water and 2 ml of dinitrophenylhydrazine solution. The resulting solution was placed in a hot water bath at 37°C for 10 min, then 2 ml of 1.5 M sodium hydroxide was added to each tube. After adding NaOH, the samples must be read quickly. The absorption of the samples at a wavelength of 515 nm was read with a spectrophotometer (Cary 100 model, manufactured by Varian Company, USA). Sodium pyruvate was also used as a standard in the concentration range of 0–60 mM instead of garlic extract (Gruhlke et al., 2019; Reiter et al., 2020).

### 2.3 Optimization by RSM

The effect of three independent variables (dose, storage time, and temperature) was investigated by Box Behnken design on the dependent variables (humidity, weight loss, color, and allicin). Assumed that there are three mathematical functions fk for yk which are in equation (1):

(1) $$y_{k}=f_{k}\;\left(\varepsilon_{1},\varepsilon_{2},\varepsilon_{3}\right)$$

where, ε_1_, ε_2_, and ε_3_ are natural variables. ε_1_, ε_2_, and ε_3_ are dose, temperature, and storage time, respectively. In the response surface method, natural variables change to coded variables (x_1_, x_2_, …) equation (2):

(2) $$\mathrm{yk}=\mathrm{fk}\;\left(x_{1},x_{2},x_{3},\ldots \right)$$

This research used a quadratic model for modeling equation (3) (Sukumar & Athmaselvi, 2019):

(3) $$y_{k}=\beta_{0}+\sum_{i=1}^{2}\beta_{i}x_{i}+\sum_{i=1}^{2}\beta_{\mathrm{ii}}x_{i}^{2}+\sum_{i=1}^{2}\sum_{j=i+1}^{2}\beta_{\mathrm{ij}}x_{i}x_{j}$$

In equation (3), y_k_ is the predicted response which was considered as dependent variables (k = 1, 2, …, 8). Xi is the input coded variable or the same independent variable (i = 1, 2, 3). The values of the independent variables were coded between −2 and + 2. All coefficients are parameters of regression coefficients. Using the quadratic model, five mathematical models were evaluated for each dependent variable. Each factor in the design was measured at three different levels (2−, 0, +2), two axial points, and six repetitions at the central point (Table 1). Experimental design as well as process optimization was performed using Design Expert11 software. Analysis of variance on quadratic model coefficients was also performed using this software. Significant sentences in the model were obtained using analysis of variance for each response.

**TABLE 1:** Independent variables and levels in model

Independent variables	Coded variables	Natural variables	Levels				
Storage time (month)	X_1_	ɛ_1_	−2	−1	0	+1	+2
Dose (Gy)	X_2_	ɛ_2_		−1	0	+1	
Temperature (°C)	X_3_	ɛ_3_		−1	0	+1	

### 2.4 Chemometrics approach

#### 2.4.1 Principal component analysis (PCA)

The principal component analysis is a statistical method that uses orthogonal transfer for the conversion of a set of observed correlated variables into a set of uncorrelated linear variables which are principal components. This conversion is performed in a way that the first component has the highest variance, and then, the other components also have high variance with limitations of course, and all components are perpendicular to the previous components. PCA is a high precession sensitive method to find main variables. PCA is one of the common methods in data analysis and dimension reduction in multivariate systems (Tazi et al., 2019). Additionally, loading plots provide information on the relative importance of this set of sensors in the analysis of principal components (Barbosa et al., 2020).

#### 2.4.2 Partial least square (PLS)

One of the most powerful techniques in the field of chemometric is factor analysis. Factor analysis is a multivariate method that provides important and useful information by reducing the size of the data and the minimum number of perpendicular vectors as PLS. PLS is a linear combination method for main variables in the matrices and instead of the I × J matrix and its variables can be defined as a linear combination of the J factor and finally, new variables are defined for the matrix (Rambo et al., 2020).

### 2.5 Mechanical parameters

#### 2.5.1 Relaxation test

In order to measure the viscoelastic properties, a relaxation test was performed on the samples in a pressure plate accordingly. A texturometer (Zwick/Roell, bt1_fr0.5th.d14 model, Germany, xforce hp load cell with 500 N capacity) with a 25‐mm‐diameter probe pressure plate was used to perform the test. These experiments were done at room temperature under the following conditions: Initial loading force 0.1 N, start speed test at 70 mm.min^−1^, load speed at 2 mm.min^−1^, and time of the test was 400 s.

The basic requirement of designing machines for the processing of fruit and food is having information about the physical characteristics of the fruit. In the lines of transportation and packing, the fruit is subjected to different loads that may damage its tissue. The stress and strain state under static and dynamic loads that are related to the nature and mechanical behavior of the material is considered as the first step in quantitative analysis of the characteristics of agricultural products (Mahiuddin et al., 2018). One of the best models for investigating load damages of fruit is the general Maxwell model stress relaxation test design. To overcome this drawback in agricultural products, springiness with coefficient E_e_ was added to the general Maxwell model (Mohsenin, 1986). Equation (4) shows the expression of Maxwell's mathematical model:

(4) $$\sigma \;\left(t\right)=\sigma_{1}.e^{\frac{-t}{\text{Trel}1}}+\sigma_{2}.e^{\frac{-t}{\text{Trel}2}}+\sigma_{e}$$

where the consideration σ_i_ = ε_0_E_i_, σ_e_ = ε_0_E_e_ and can be written as equation (5):

(5) $$\sigma \;\left(t\right)=\left[E_{1}.e^{\frac{-t}{\text{Trel}1}}+E_{2}.e^{\frac{-t}{\text{Trel}2}}+E_{e}\right]\varepsilon_{0}$$

The obtained model coefficients were determined and evaluated from relaxation stress curves. Residues were determined using the sequential model. All stress relaxation time models were calculated by MATLAB R.13 software.

#### 2.5.2 Shear test

**FIGURE 1:**
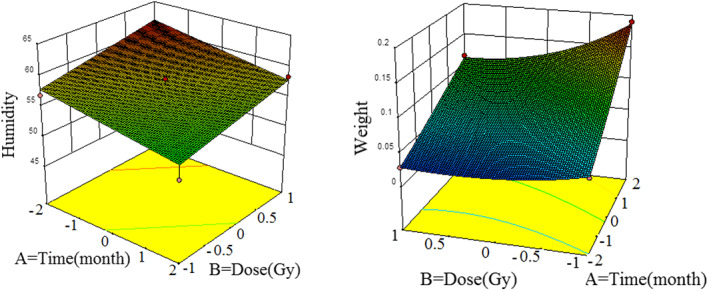
3D couture of humidity and weight loss changing with dose, time, and temperature

In order to determine enough shear force for the garlic product, an experimental shear tool with a commercial single sickle knife section and a counter shear was used as the twofold shear test. Finally, the test was done using a Zwick/Roell texturometer. The diameter cutter probe, preload, distance of probe to the bottom of the page, and speed at start position are 0.5 mm, 0.3 N, 65, mm, and 300 mm. min^−1^, respectively (Figure 1).

The shear stress was calculated in MPa using equation (6) (Mohsenin, 1986):

(6) $$\tau =\frac{F_{\max}}{A}$$

where F_max_ is the maximum shear force (N) and A is the cross‐sectional area of the stalk at shear planes (mm^2^). The shear energy was calculated by the area under these curves to the maximum force of the curve (Chen et al., 2004). The cross‐sectional area for calculating the shear test was calculated by Solid Works R.2015 software. For this purpose, garlic cloves were designed in the software to obtain the main dimensions (length, width, and thickness) of the samples. By drawing a three‐dimensional image, the rectangular area was measured in these dimensions (Figure 1).

### 2.6 Statistical analysis

These experiments were designed based on a factorial design with three factors of gamma ray (control, 75, and 150 Gy doses), storage time (1–5 months), and the storage temperature (4 and 18°C). The analysis of variance was carried out on data using SPSS software (IBM SPSS Statistics 22, IBM, NY). Also, differences between means were determined with Tukey post hoc comparison tests. *p*‐values of 0.05 or less were considered significant.

## 3 RESULTS AND DISCUSSION

### 3.1 Optimization

According to the results of (Figure 1), the effects of radiation dose and storage time variables were significant on humidity variables. With a 150 Gy irradiation dose and 5 months of storage time, the lowest amount of humidity was observed in garlic samples. With increasing storage time, the humidity in the control and irradiated samples of 75 and 150 Gy decreased to 14.51%, 17.37%, and 18.97%, respectively. Also, with increasing the intensity of irradiation, the percentage of humidity in irradiated samples with intensities of 75 and 150 Gy decreased to 4.15% and 6.94%, respectively, compared to the control sample at the end of storage. The amount of initial humidity indicates water loss of the product and water loss mainly depends on the intensity of product respiration and ethylene production (Sukumar & Athmaselvi, 2019).

**FIGURE 2:**
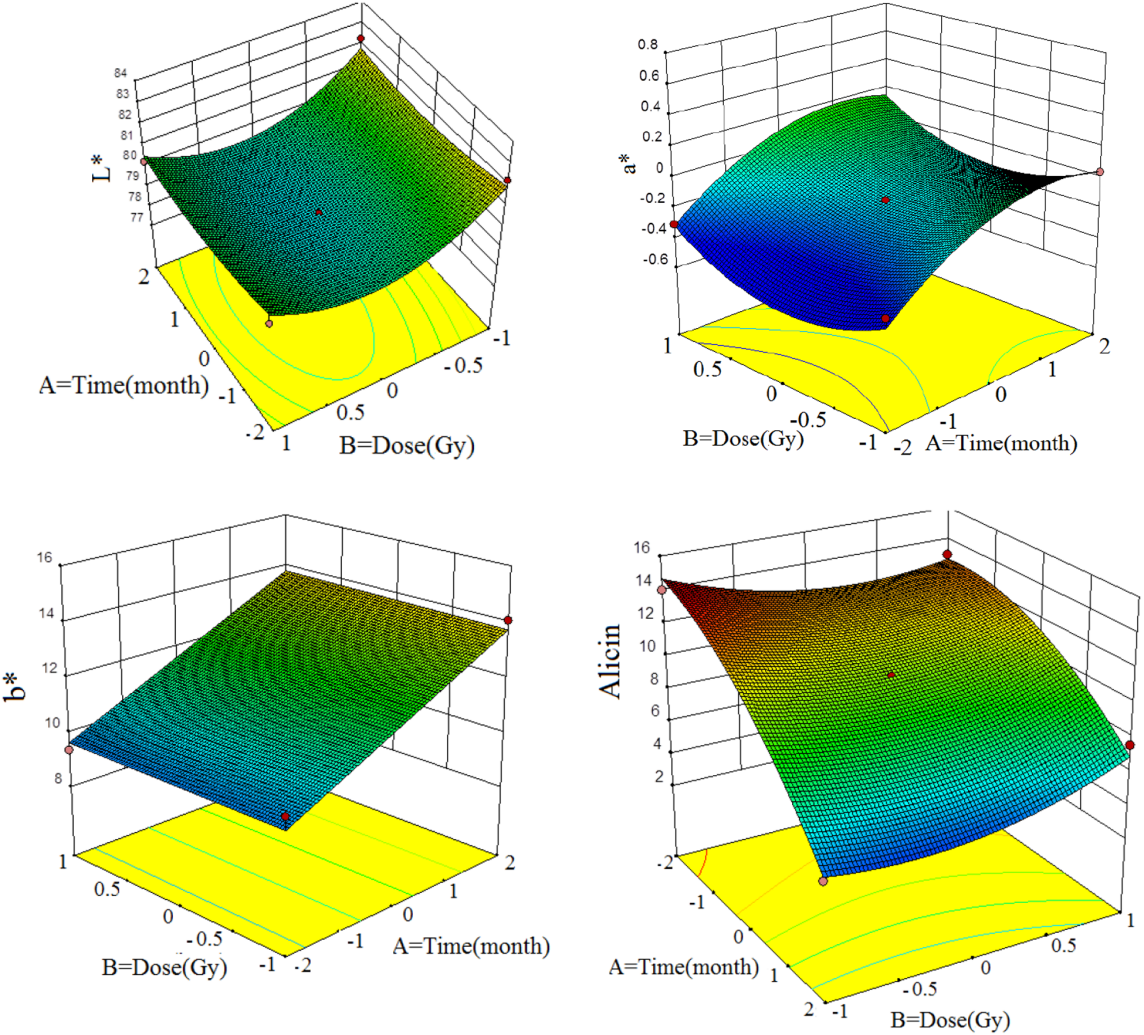
3D couture of color parameters and allicin changing with dose, time, and temperature

Also, (Figure 1) shows weight loss changes after 5 months of storage and the high effect of 150 Gy dose on garlic samples. According to the results, storage time is a very important and influential indicator on weight loss. Over the storage time and increasing the intensity of irradiation, the percentage of weight loss increased significantly during 5 months. Weight loss will be followed by dehydration, metabolic activity, respiration, and transpiration (Ghasemi & Chayjan, 2019). Respiration intensity in fruits and vegetables increases with ripening, which reduces the storage of nutrients in products and weight loss (Durante et al., 2020). Also, the application of radiation, first with the application of 75 Gy intensity and then 150 Gy intensity increased the percentage of weight loss (Figure 2). One of the most important factors in weight loss during irradiation is water loss due to energy absorption caused by waves (Sukumar & Athmaselvi, 2019). In a study, the effect of storage time for 6 months in cold and traditional warehouses on garlic cultivars in Hamadan was investigated. According to the results, with increasing storage time, the percentage of weight loss of samples increased and in cold warehouses, these changes were less than traditional warehouses (Tazi et al., 2019). Another study was investigated the effect of radiation intensity of 50 and 150 Gy and storage time for 45 days on garlic samples. According to the results, with increasing storage time, the percentage of weight loss increased and irradiated samples had a higher percentage of weight loss than the control (Sukumar & Athmaselvi, 2019).

Analysis of variance (Table 2) on humidity and weight loss indices is presented the effect of independent variables of radiation dose, storage temperature, and time. Statistically, it is a significant model with a *p*‐value of less than 0.05. Based on the results of *p*‐value in modeling, the humidity content of the sample is equal to 0.0144 and shows a significant effect of independent variables on humidity. Also, the main effects of three independent parameters of time and temperature on humidity are significant. Also, the main and secondary effects of independent variables of A, B, and C on weight loss are significant.

**TABLE 2:** Analysis of variance of humidity based on dose, time, and temperature

Parameter	Source	Sum of squares	Df	Mean square	F value	*p*‐value
						Prob > F
Humidity	Model	0.42	3	0.14	3.37	0.0414
	A‐Time	0.13	1	0.13	3.20	0.0467
	B‐Dose	0.13	1	0.13	3.16	0.0488
	C‐Temp	0.16	1	0.16	3.76	0.0747
	Residual	0.54	13	0.042		
	Lack of Fit	0.054	9	0.060		
	Pure Error	0.000	4	0.000		
	Cor Total	0.97	16			
Weight loss	Model	0.098	9	0.011	238.32	<0.0001
	A‐Time	0.072	1	0.072	1570.36	<0.0001
	B‐Dose	0.011	1	0.011	232.97	<0.0001
	C‐Temp	1.826 E‐003	1	1.826 E‐003	39.94	0.0004
	AB	6.074 E‐004	1	6.074 E‐004	13.28	0.0082
	AC	6.702 E‐004	1	6.702 E‐004	14.66	0.0065
	BC	7.957 E‐003	1	7.957 E‐003	174.03	<0.0001
	A^2^	5.218 E‐004	1	5.218 E‐004	11.41	0.0118
	B^2^	2.804 E‐003	1	2.804 E‐003	61.32	0.0001
	C^2^	1.466 E‐003	1	1.466 E‐003	32.06	0.0008
	Residual	3.200 E‐004	7	4.572 E‐005		
	Lack of Fit	3.200 E‐004	3	1.067 E‐004		
	Pure Error	0.000	4	0.000		
	Cor Total	0.098	16			

Changes in the brightness of garlic samples (Figure 2) at the beginning of storage or in the early months of storage have higher values and decreased the brightness of the samples after 5 months. According to the results of the analysis of variance (Table 3), the storage time on the L* index was not significant but had a great effect on the main effects of dose and temperature on the L* sample. Among the interactions, the interaction of the AB parameter on L* was not significant but the effects of AC and BC on L* were significant. The results of (Figure 2) show the three‐dimensional contour L*. According to the results, the control sample and 150 Gy had higher brightness.

**TABLE 3:** Analysis of variance of L*, a*, and b* based on dose, time, and temperature

Parameter	Source	Sum of squares	Df	Mean square	F value	*p*‐value
						Prob > F
L*	Model	31.40	9	3.49	13.86	0.0011
	A‐Time	0.074	1	0.074	0.29	0.6042
	B‐Dose	3.25	1	3.25	12.92	0.0088
	C‐Temp	11.26	1	11.26	44.74	0.0003
	AB	4.225 E‐003	1	4.225 E‐003	0.017	0.9005
	AC	1.14	1	1.14	4.55	0.0704
	BC	1.33	1	1.33	5.30	0.0548
	A^2^	0.79	1	0.79	3.13	0.1202
	B^2^	11.88	1	11.88	47.22	0.0002
	C^2^	0.81	1	0.81	3.20	0.1166
	Residual	1.76	7	0.25		
	Lack of Fit	1.76	3	0.59		
	Pure Error	0.000	4	0.000		
	Cor Total	33.16	16			
a*	Model	1.30	9	0.14	20.60	0.0003
	A‐Time	0.43	1	0.43	61.68	0.0001
	B‐Dose	0.021	1	0.021	3.00	0.1270
	C‐Temp	5.513 E‐003	1	5.513 E‐003	0.79	0.4047
	AB	0.000	1	0.000	0.000	1.0000
	AC	0.036	1	0.036	5.15	0.0575
	BC	0.099	1	0.099	14.15	0.0071
	A^2^	0.18	1	0.18	26.17	0.0014
	B^2^	0.21	1	0.21	30.07	0.0009
	C^2^	0.33	1	0.33	46.67	0.0002
	Residual	0.049	7	7.011 E‐003		
	Lack of Fit	0.049	3	0.016		
	Pure Error	0.000	4	0.000		
	Cor Total	1.35	16			
b*	Model	39.47	6	6.58	24.91	<0.0001
	A‐Time	34.07	1	34.07	129.01	<0.0001
	B‐Dose	8.000 E‐004	1	8.000 E‐004	3.029 E‐003	0.9572
	C‐Temp	0.60	1	0.60	2.27	0.1628
	AB	1.225 E‐003	1	1.225 E‐003	4.638 E‐003	0.9470
	AC	4.62	1	4.62	17.50	0.0019
	BC	0.17	1	0.17	0.65	0.4382
	Residual	2.64	10	0.26		
	Lack of Fit	2.64	6	0.44		
	Pure Error	0.000	4	0.000		
	Cor Total	42.11	16			
Allicin	Model	155.59	9	17.29	37.02	<0.0001
	A‐Time	126.69	1	126.69	271.34	<0.0001
	B‐Dose	0.067	1	0.067	0.14	0.7156
	C‐Temp	0.55	1	0.55	1.17	0.3154
	AB	4.40	1	4.40	9.43	0.0180
	AC	2.06	1	2.06	4.42	0.0736
	BC	0.46	1	0.46	0.99	0.3539
	A^2^	12.64	1	12.64	27.07	0.0012
	B^2^	7.12	1	7.12	15.24	0.0059
	C^2^	2.69	1	2.69	5.76	0.0475
	Residual	3.27	7	0.47		
	Lack of Fit	3.27	3	1.09		
	Pure Error	0.000	4	0.000		
	Cor Total	158.86	16			

According to the analysis of variance (Table 3), the main effect of storage time on the parameter a* is significant and the main effects of B and C on a* are not significant. Also, the interactions of AC and BC on a* are significant. The effects of the square on a* are significant. According to (Figure 2), in the control sample and 150 Gy, a* is more than in the 75 Gy sample.

With increasing storage time, the value of a* increased, which is due to the reduction of humidity in the sample tissue and the shrinkage process (Kainthola et al., 2020). With increasing the amount of radiation intensity, the value of a* increased compared to nonirradiated samples. Because, as mentioned, with increasing the intensity of radiation and absorption of this energy by intracellular water molecules and its evaporation, the intracellular water decreases and the amount of redness increases over time (Mohammad‐Razdari et al., 2021).

According to the results of analysis of variance (Table 3), the main effect of time on b* is significant and the main effects of dose and temperature on b* were not significant. Also, among the interactions, only AC is significant. Due to the three‐dimensional contour (Figure 2), the dose parameter is ineffective and only has increased b* in all samples after 5 months.

On the other hand, with increasing storage time, b* or the amount of jaundice increased and with the application of irradiation intensity, b* of the irradiated sample decreased by 75 Gy compared to the control sample but increased the amount of b*with increasing irradiation intensity up to 150 Gy. Increased b* with increasing radiation intensity is due to the fact that most plant products or organs typically lose large amounts of volatiles during puberty and aging. The yellowing of the white garlic sample also has a linear relationship with the time elapsed after harvest, which is also proportional to the intensity used (Olanya et al., 2015). In a study, by increasing the amount of radiation intensity to a certain value and putting the samples at 4°C, the amount of b* of the irradiated sample increased less than the control sample (Durante et al., 2020). This means that the L* index decreases with increasing storage time and increasing the amount of irradiation intensity (Tables 1 and 2). This is due to the decrease in humidity and the reduction of water between cells during the storage period (Noda et al., 2018). On the other hand, with increasing radiation intensity, the intracellular water, as mentioned above, decreases and evaporation takes place (Sukumar & Athmaselvi, 2019). Similar results were obtained in another study that decreased the value of L* with increasing the amount of radiation intensity and storage time (Durante et al., 2020).

Table 4 shows the results of RSM analysis of variance and the proposed model. According to the results, there are significant changes in storage time on allicin. But the main changes in irradiation dose and storage temperature on the allicin are not significant. Also, among the interactions, the interaction effect of storage time at irradiation dose is significant on allicin. But the quadratic effects of all independent variables (time–dose and temperature) on allicin are significant. According to the three‐dimensional contour (Figure 2), it can be said that at the beginning of storage, the allicin was high, but after 5 months, the allicin has decreased sharply, and this reduction has occurred in all samples and treatments.

**TABLE 4:** Optimization equations with coefficients and *p*‐value for model

Response	Intercept	A	B	C	AB	AC	BC	A^2^	B^2^	C^2^
Humidity	7.6009	−0.1294	0.1285	0.1401						
P		0.0467	0.0488	0.0747						
Weight Loss	0.2747	0.0947	−0.0364	−0.0151	−0.012	0.0129	−0.0446	−0.0111	0.0258	−0.0186
P		<0.0001	<0.0001	0.0004	0.008	0.0065	<0.000	0.0118	0.0001	0.0008
L	79.15	−0.09625	−0.6375	−1.18625	−0.0325	−0.535	0.5775	0.4325	1.68	0.4375
P		0.6042	0.0088	0.0003	0.9005	0.0704	0.0548	0.1202	0.0002	0.1166
a*	−0.15	0.2325	0.05125	0.02625	−0.0000	−0.095	0.1575	−0.20875	0.2237	0.27875
P		0.0001	0.1270	0.4047	1.0000	0.0575	0.0071	0.0014	0.0009	0.0002
b*	11.7194	2.06375	0.01	0.27375	0.0175	1.075	−0.2075			
P		<0.0001	0.9572	0.1628	0.9470	0.0019	0.4382			
Allicin	10.1767	−3.97954	0.0916	−0.2612	1.0491	−0.7184	−0.3391	−1.73254	1.3000	0.799125
P		<0.0001	0.7156	0.3154	0.0180	0.0736	0.3539	0.0012	0.0059	0.0475
Legend		*p* < .01	.01 <= *p* < .05	.05 <= *p* < .10	*p* >= .10					

As the shelf life increased, the stiffness of the samples decreased (Figure 2). Measurement of pyruvate during 5 months of storage shows the enzymatic degradation and decomposition of allicin, which results in a reduction in the aroma and flavoring compositions of garlic during storage. On the other hand, for irradiated samples, the intensity of allicin decrease was less. This means that by irradiating the samples, the allicin is maintained and the gamma ray prevents the decomposition and degradation of enzymatic compounds, and with increasing intensity, further degradation of enzymes is prevented (Chen et al., 2020). In one study, a decrease in pyruvate was observed during storage, which is due to the breakdown of tasteless compounds by peptidase enzymes and in fact reduces the activity of the enzyme alliinase and the aroma and flavoring compounds of garlic (Chen et al., 2020; Mandal et al., 2019).

### 3.2 Introducing optimal points and desirability index

**FIGURE 3:**
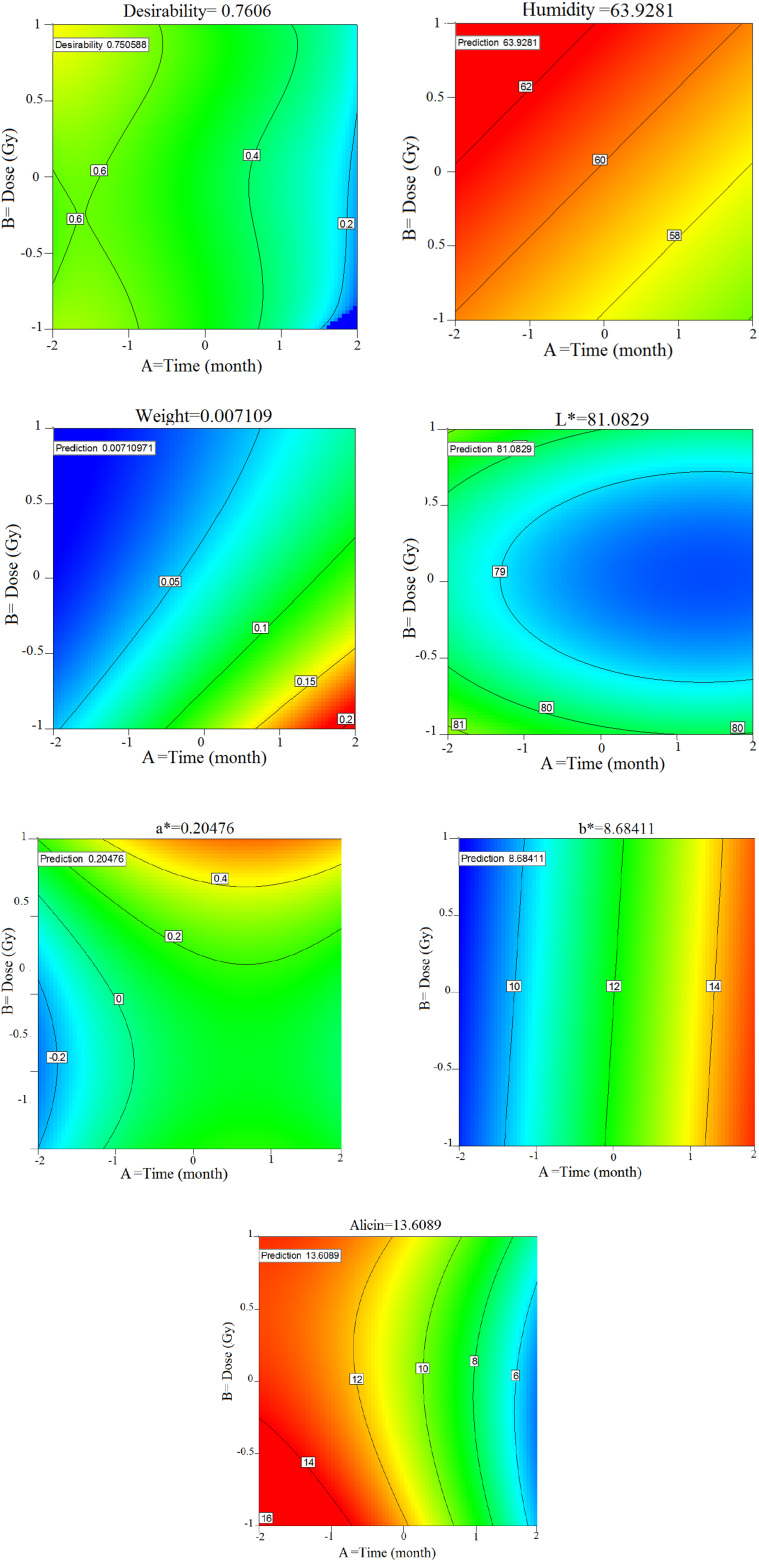
2D couture of desirability, color parameters, and allicin changing with dose, time, and temperature and best optimal point

Based on the results of the desirability index, the best and most optimal answer is obtained for the variables of time, temperature, and dose so that it can be decided based on the results of the variables of humidity, weight loss, color, and allicin to maintain garlic samples. Desirability index is the way to find the best points that are 0.76. According to (Figure 3), the desirability index had the highest value at the beginning of the storage period. According to the contour of (Figure 3), the humidity was high at the beginning of storage time and decreased after 5 months. The best humidity point for garlic samples is 63.9281. Also, there is weight loss in irradiated samples and control during storage. Proper storage of garlic products should have an optimal value of 0.0071 weight loss. Moreover, according to the contour results, the best values of L*, a*, b*, and allicin are 81.082, 0.2047, 8.6841, 8.7033, and 13.6084, respectively, and the best storage temperature is 17°C.

According to the results and expressions of quadratic equations, there is a linear relationship between dependent and independent variables. The positive sign of the coefficients indicates the direct effect and the negative sign indicates the inverse effect of the independent and dependent variables. According to (Table 4), for the humidity‐dependent variable, the intercept is 7.6009 and the negative coefficients A indicate that the storage time has an inverse effect on humidity and humidity decreases with increasing storage time. According to the equations, the increase of A, AC, and B2 decreases B, C, AB, BC, and A2 variables, and C2 increases weight loss. Also for the variable L*, the increase of A, B, C, AB, and AC decreases L* and the BC, A2, B2, and C2 variables are directly related to L*. According to (Table 4), A, B, C, BC, B2, and C2 parameters are directly related to a* and have reverse relation to the other parameters. Also, all parameters except BC are directly related to b* and as these parameters increase, b* also increases.

Finally, in the study of the degree of allicin, the independent parameters A, C, AC, BC, and A^2^ have an inverse effect on the allicin and if these parameters increase independently, the reverse downward trend occurs for the allicin and the parameters B, AB, B^2^, and C^2^ are also related. It has a steep downward trend and increases allicin with their increase.

### 3.3 Classification of weight loss by PCA

**FIGURE 4:**
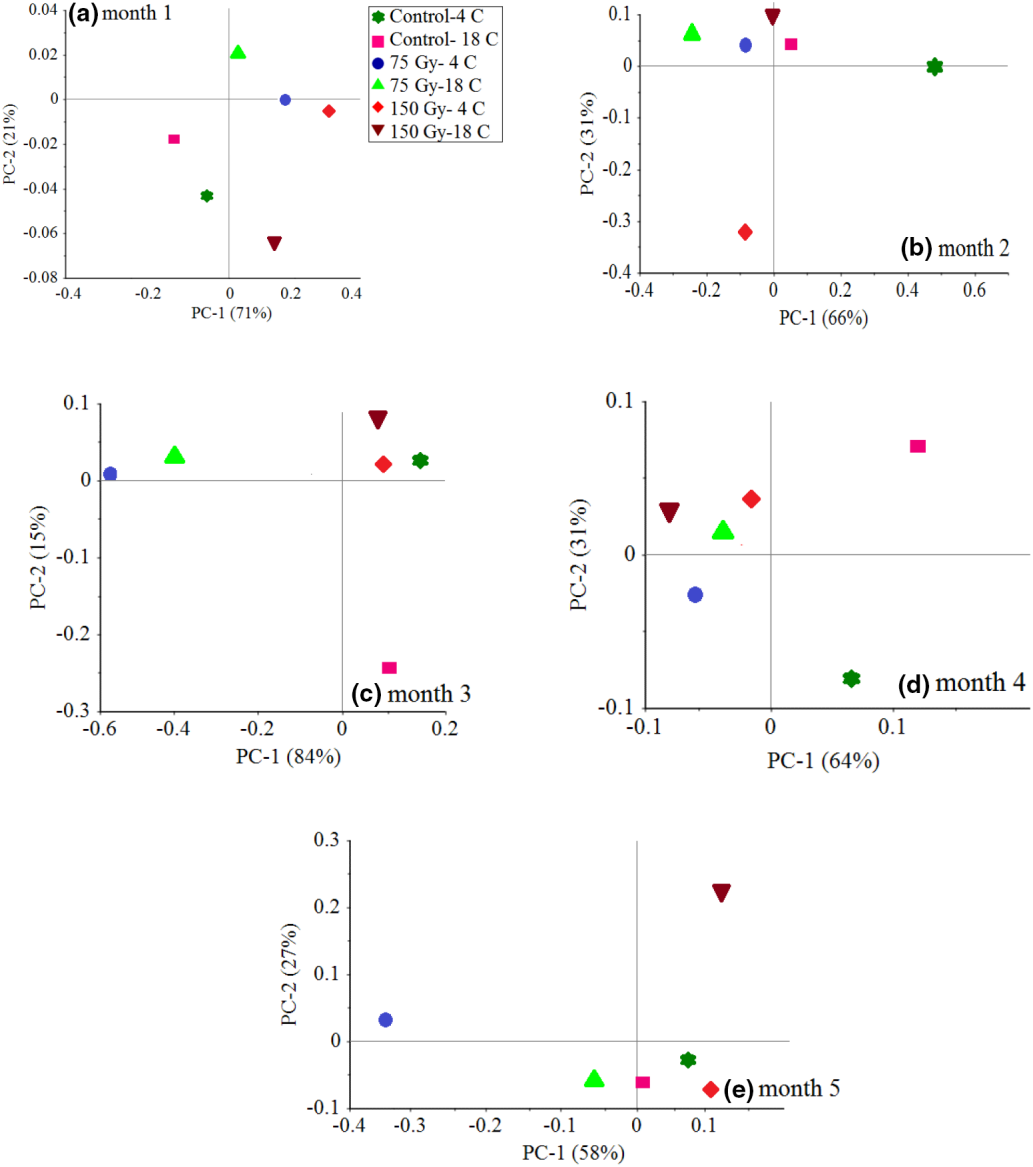
PCA classification for control and irradiated samples in 5‐month storage based on storage time (a) month 1, (b) month 2, (c) month 3, (d) month 4, and (e) month 5

Due to the large effect of independent variables on weight loss, PCA results of weight loss changes have been investigated. The results of PCA on weight loss show a good and significant difference between the control samples, 75 and 150 Gy at two temperatures of 4 and 18°C and in different months. (Figure 4a) of PC1 and PC2 covers the ability of 71% and 21% of the data, respectively, and observed a total of 92% of the variance between the data, and is able to make a good distinction between the samples. It is noteworthy that the control groups, 75 and 150 Gy, have been identified together and can be grouped together. In the second month of storage, PC1 and PC2 were able to distinguish 66% and 31% of the variance between the data and 97% of the total variance between the data, respectively, and were able to differentiate the data. This indicates the high accuracy during testing and the ability of PC detection in data classification. Also in months 3, 4, and 5, PC values have 99%, 95%, and 85% variance between data, respectively.

**FIGURE 5:**
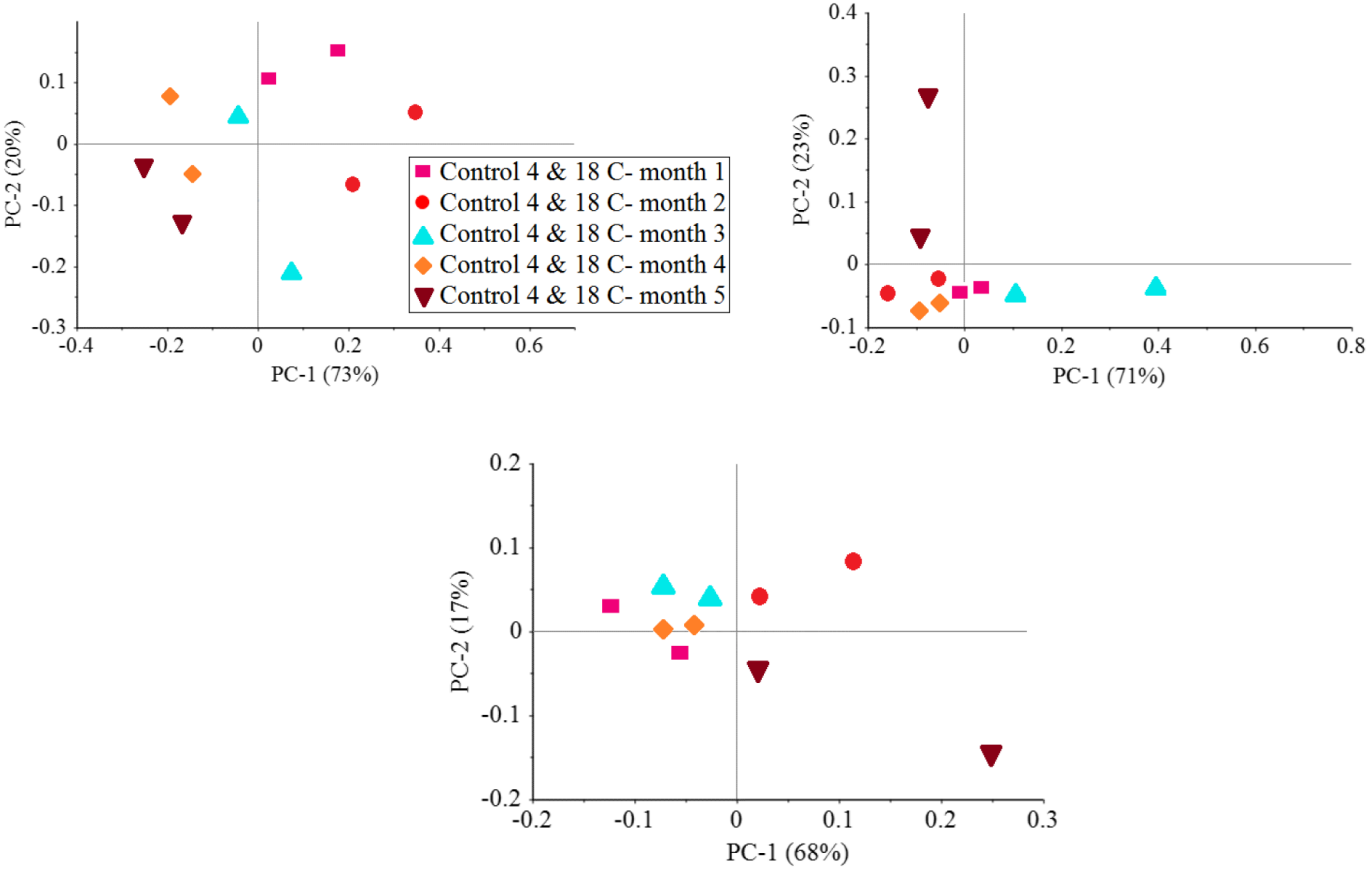
PCA classification for control and irradiated samples in 5‐month storage based on treatments

The best classification was done in month 3. In other words, it can be said that from month 3 onwards and with the aging process of garlic samples, the data in almost all groups are closer to each other and the weight loss of samples is obvious. Also, (Figure 5) shows the PC results for separating the samples at 5 months of storage for the control samples, 75 and 150 Gy. PC was able to cover a total of 93% of the variance between the data and in the samples of irradiated garlic with a dose of 75 and 150 Gy, the PC was able to cover 94% and 85% of the variance between the data and it can be said that the overall optimization results with RSM and high PCA accuracy, the best radiation dose is 75 Gy to see the best weight loss results.

### 3.4 Prediction by PLS

**FIGURE 6:**
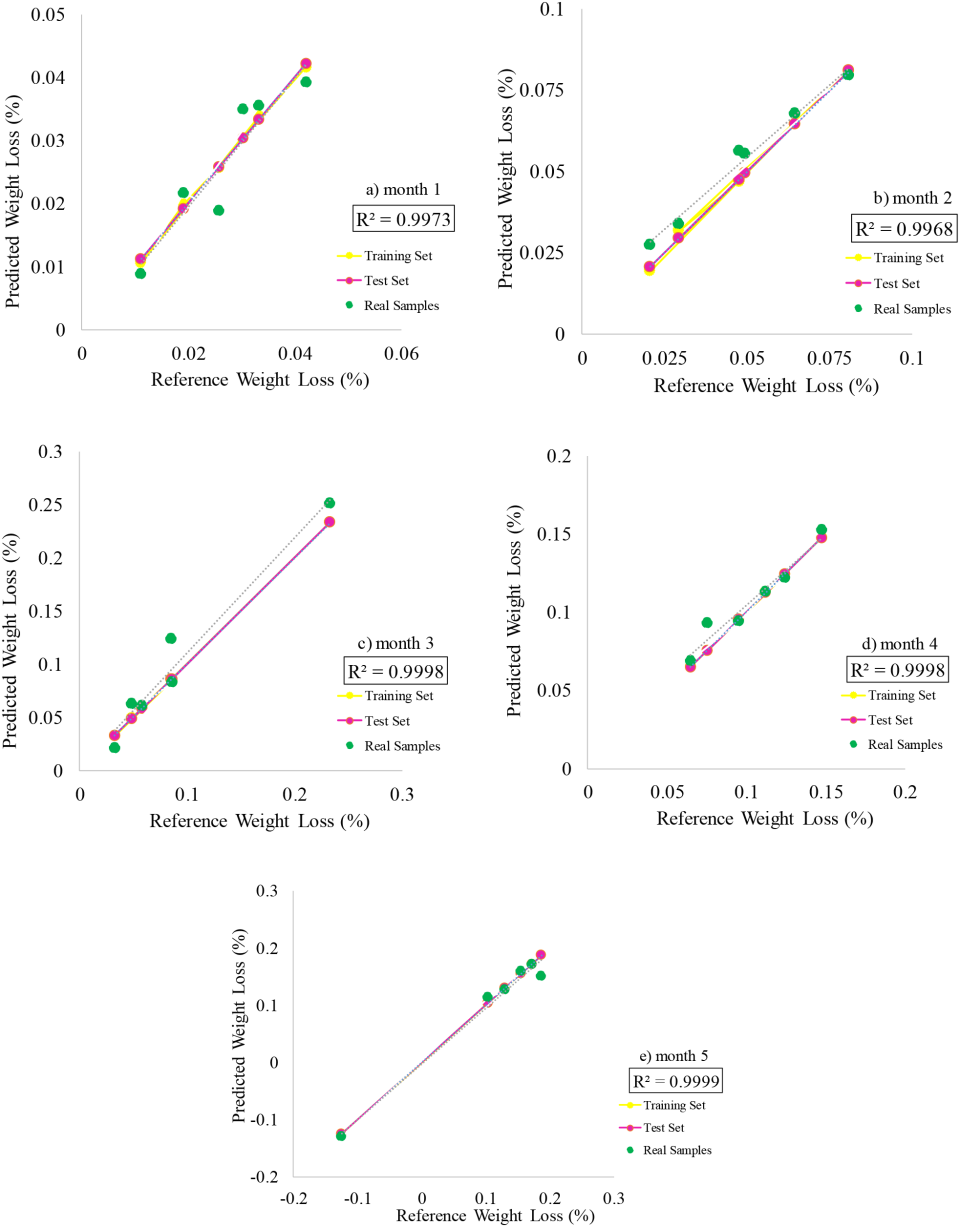
PLS results for weight loss of all treatments

In this section, (Figure 6) shows the PLS results of weight loss data in the control and irradiated samples at 5 months of storage at 4 and 18°C. Predicted weights versus actual weights are shown and the results show that PLS has the ability to predict data in 5 months of high‐performance R^2^ storage.

### 3.5 Relaxation test

Table 5 shows the mean values of stress and relaxation time in the viscoelastic element for the two‐component Maxwell model that is calculated with equation (1). MATLAB R.13 software was used to fit data on the models. In this way, with the insertion of variables, the best model and relaxation time was concluded with CF Tool command and definition of stress function. According to the result, relaxation time increased with increasing storage time and this process was greater for the irradiated samples than for control samples. Also, the relaxation time for samples placed at 18°C was 27% longer than samples placed at 4°C. The results showed that irradiation caused humidity loss, and samples lose their viscous state and become more elastic (Calado et al., 2018).

**TABLE 5:** Summary of analysis of variance of Maxwell element in a factorial experiment

Dose treatment (Gy)	Temperature (°C)	Stress (MPa)			Relaxation time (s)	
		σ_1_	σ_2_	σ_e_	T_rel1_	T_rel2_
150	4	5.0112	5.2523	4.9878	1 × 10^−6^	11 × 10^−4^
	18	4.8796	5.1121	4.8318	3 × 10^−7^	11 × 10^−5^
75	4	4.8983	5.117	4.90	4 × 10^−7^	17 × 10^−5^
	18	4.9293	5.1460	5.11	6 × 10^−8^	2 × 10^−6^
0	4	4.8874	5.1178	4.8873	6 × 10^−8^	11 × 10^−6^
	18	4.94	5.092	4.9426	8 × 10^−8^	8 × 10^−6^

Relaxation time is different based on the characteristics of the viscoelastic or viscous substances, but this time is longer in elastic material. The results of chemical studies (Calado et al., 2018) showed that irradiation is the cause of humidity loss in the product and evaporation of water within tissue. With increasing storage time, the relaxation time average in the control and irradiated samples with 75 and 150 Gy doses are 5%, 9%, and 12% at 4°C and 8.5%, 11%, and 17% at 18°C, respectively. The relaxation time depends on the humidity content of the product and is reduced because of the humidity (Danalache et al., 2015). However, critical stress decreases with an increase in storage time, but this change does not occur in irradiated samples. Thus, irradiated garlic samples with 150 Gy dose have more critical stress too.

### 3.6 Shear test

**FIGURE 7:**
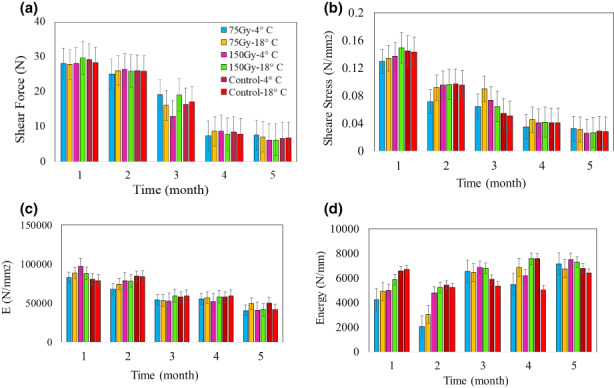
Effect of storage time, irradiation dose, and temperature on (a) shear force, (b) shear stress (c) Young's modulus, and (d) energy of rupture

As shown in Figure 7a, shear stress in garlic is reduced with increasing storage time, but irradiation keeps the shear stress almost constant. According to the results, shear stress was reduced by about 27%, 23%, and 18% at 4°C for control samples and 32%, 29%, and 24% at 18°C for irradiated samples (75 and 150 Gy). Lower temperature (4°C) does not allow the shear stress to reduce, and as a result, irradiated and control samples stored at 4°C have higher values of shear stress.

Shear strain was significantly reduced, because of the increase in storage time and storage temperature (Figure 7b). But in contrast, irradiation prevents shear strain reduction, because of the tissue damage and softening of the stored sample (Calado et al., 2018). As shear strain in control, irradiated with 75 and 150 Gy doses decreased 29%, 23%, and 20% at 4°C and 35%, 28%, and 26% at 18°C, respectively.

According to the results (Table 6), energy of rupture reduced with increasing storage time and storage temperature. But, when increased irradiation dose, the energy of rupture has less reduction, because of the preservation of tissues from penetration. Thus, the values for control and irradiated samples were (75 and 150 Gy) 14%, 13%, and 13% for storage at 4°C, and 17%, 16%, and 15% for storage at 18°C, respectively. Figure 7c shows the preserved tissues in irradiated samples and they have higher values.

**TABLE 6:** Analysis of variance results for main effect of irradiation, temperature, storage, and their interaction on measured parameters

Variables	Df	τ_max_ (MPa)	ɛ_max_	E_max_ (MPa)	σ_rel_ (MPa)	T_rel_
Irradiation	2	0.01523^a^	0.2213^a^	0.001025^a^	0.00^b^	0.011^a^
Temperature	1	0.02136^a^	0.1145^a^	0.005621^a^	0.00^b^	0.498^a^
Storage	5	0.345^c^	0.223^c^	0.528^c^	0.0131^a^	0.711^c^
Irradiation × temperature	2	0.02232^b^	0.1256^b^	0.01002^a^	0.0311^a^	0.0304^a^
Irradiation × storage	10	0.02136^a^	0.1145^a^	0.005621^a^	0.601^c^	0.609^c^
Temperature × storage	5	0.01964^b^	0.1056^a^	0.003216^b^	0.192^c^	0.457^c^
Irradiation × temperature × storage	10	0.01687^a^	0.0451^b^	0.006321^a^	0.122^c^	0.647^c^
Test error	41	0.001247	0.0225	0.2654	0.06254	1.012
CV (%)		9.52	3.254	0.987	21.45	2.365

^a^ Significant relationship between two parameters at *p* < .05.

^b^ Significant relationship between two parameters at *p* < .01.

^c^ Means there was no significant relationship here.

As mentioned, increasing storage time can decrease the shear stress, because the garlic samples lose their intracellular water with increasing storage time. Of course, due to biological activity, breathing decreased humidity content (Pérez et al., 2007). Compressibility is most of the original strains because the cells are full of water in fresh garlic (Zhou et al., 2010). In a study that had been done on garlic, there was a decrease in the shear stress and energy of rupture when tissue was destroyed (Llamas et al., 2013).

## 4 CONCLUSIONS

Irradiation preserves mechanical and physical properties of the food. In this study, irradiated samples stored at 18 and 4°C had lower changes in color parameters (a*, b*, and L*). Also, based on the results, irradiation has a significant effect on garlic tissues and preserves the tissues of the sample, but a high dose has the reverse effect on shear stress, shear strain, and energy of rupture. Therefore, the storage time had a significant effect on mechanical properties. Likewise, results showed that chemometrics methods like PCA and PLS can classify and predict storage condition data exactly. Then, in a relaxation test, two components of the Maxwell model were obtained for garlic cloves. Moreover, with increasing storage time, the relaxation time average increased in irradiated samples than in the control samples. Finally, the results showed that the irradiation preserved some mechanical and physical properties of garlic during 5 months of storage.

## FUNDING INFORMATION

This study was supported by the office of vice chancellor for research at Bu‐Ali Sina University (Thesis No. 4375).

## CONFLICT OF INTEREST

The authors have no conflict of interest to declare.

## PRACTICAL APPLICATIONS

Irradiation is an effective way for increasing the shelf life of food products. Irradiated garlic stored for 5 months under controlled condition and optimization of effective parameters was done by RSM. Also, discrimination of irradiated garlic was done by chemometric approaches. Moreover, a mechanical test was completed for the storage simulation by the General Maxwell model and shear test.

## Data Availability

The data that support the findings of this study are available on request from the corresponding author.
